# Evaluating machine learning-powered classification algorithms which utilize variants in the GCKR gene to predict metabolic syndrome: Tehran Cardio-metabolic Genetics Study

**DOI:** 10.1186/s12967-022-03349-z

**Published:** 2022-04-09

**Authors:** Mahdi Akbarzadeh, Nadia Alipour, Hamed Moheimani, Asieh Sadat Zahedi, Firoozeh Hosseini-Esfahani, Hossein Lanjanian, Fereidoun Azizi, Maryam S. Daneshpour

**Affiliations:** 1 Biostatistics, Cellular and Molecular Research Center, Research Institute for Endocrine Sciences, Shahid Beheshti University of Medical Sciences, Tehran, Iran; 2 Biostatistics, Department of Biostatistics, Faculty of Medical Sciences, Tarbiat Modares University, Tehran, Iran; 3 Department of Surgery, University of Pittsburgh, PA, USA; 4 Cellular and Molecular Research Center, Research Institute for Endocrine Sciences, Shahid Beheshti University of Medical Sciences, Tehran, Iran; 5 Nutrition and Endocrine Research Centre, Research Institute for Endocrine Sciences, Shahid Beheshti University of Medical Sciences, Tehran, Iran; 6 Endocrine Research Center, Research Institute for Endocrine Sciences, Shahid Beheshti University of Medical Sciences, Tehran, Iran

**Keywords:** Decision tree, Discriminant analysis, Logistic Regression, Metabolic syndrome, Random Forest, Support vector machines

## Abstract

**Background:**

Metabolic syndrome (MetS) is a prevalent multifactorial disorder that can increase the risk of developing diabetes, cardiovascular diseases, and cancer. We aimed to compare different machine learning classification methods in predicting metabolic syndrome status as well as identifying influential genetic or environmental risk factors.

**Methods:**

This candidate gene study was conducted on 4756 eligible participants from the Tehran Cardio-metabolic Genetic study (TCGS). We compared predictive models using logistic regression (LR), Random Forest (RF), decision tree (DT), support vector machines (SVM), and discriminant analyses. Demographic and clinical features, as well as variables regarding common GCKR gene polymorphisms, were included in the models. We used a 10-repeated tenfold cross-validation to evaluate model performance.

**Results:**

50.6% of participants had MetS. MetS was significantly associated with age, gender, schooling years, BMI, physical activity, rs780094, and rs780093 (P < 0.05) as indicated by LR. RF showed the best performance overall (AUC-ROC = 0.804, AUC-PR = 0.776, and Accuracy = 0.743) and indicated BMI, physical activity, and age to be the most influential model features. According to the DT, a person with BMI < 24 and physical activity < 8.8 possesses a 4% chance for MetS. In contrast, a person with BMI ≥ 25, physical activity < 2.7, and age ≥ 33, has 77% probability of suffering from MetS.

**Conclusion:**

Our findings indicated that, on average, machine learning models outperformed conventional statistical approaches for patient classification. These well-performing models may be used to develop future support systems that use a variety of data sources to identify persons at high risk of getting MetS.

**Supplementary Information:**

The online version contains supplementary material available at 10.1186/s12967-022-03349-z.

## Introduction

The metabolic syndrome (MetS) refers to the simultaneous occurrence of a set of interrelated factors (high blood sugar, high blood pressure, dysregulated blood lipids, and abdominal obesity) that increases the risk of cardiovascular diseases (CVD), type 2 diabetes (T2D) and different types of cancer [1]. The pathophysiological mechanisms behind the MetS are complex and involve genetic and environmental factors, such as lifestyle, diet, and physical inactivity [2].

The prevalence of the MetS is reported to be about 31% worldwide. While this number varies between genders and ethnicities, MetS is generally more prevalent among men and women of nations with aging populations [3, 4]. In Iran, approximately 33.7% of adults suffer from this syndrome [5]. As the rising prevalence of MetS among the aging Iranian population can lead to higher CVD rates and other devastating diseases, this affliction demands further investigative efforts in all aspects [6–8].

Designing predictive models that can aid in diagnosing patients who are more likely to have MetS, can aid preventive interventions designed to battle this syndrome as well as future cardiovascular complications that it engenders. While researchers have frequently been using clinical or demographic variables in these efforts, developing models that incorporate genetic variables is complex. As MetS is a complicated multifactorial disease, taking advantage of the data on well-researched MetS-associated genes has the potential to provide us with much more powerful predictive tools.

Glucokinase (GCK) enzyme is the primary glucose sensor in the liver and pancreatic cells. It regulates carbohydrate metabolism by adjusting biochemical pathways in glycogen synthesis, gluconeogenesis, and insulin release by pancreatic β-cells. Glucokinase regulatory protein (GKRP) binds to glucokinase and controls its intracellular location and activity. The glucokinase regulator (GCKR) gene resides on the short arm of chromosome 2 (2p23.3-p23.2), contains 19 exons, and encodes GKRP (68 kDa, 625 amino acids) [9, 10]. Genome-wide association studies (GWAS) and multiple candidate gene studies have reported GCKR variants to be associated with several metabolic parameters, including triglyceride (TG) levels [11–16], insulin resistance and fasting plasma glucose (FPG) levels [14, 15, 17] as well as metabolic disorders like T2DM [12, 15, 17], dyslipidemia (high TG and low high-density lipoprotein (HDL) cholesterol levels) [11, 13]. Common functional variants, rs780094, rs780093, and rs1260326, are the most researched genetic variants of the GCKR gene. Minor T-alleles of rs780094 and rs1260326 are linked to hypertriglyceridemia, lower insulin resistance, and plasma glucose levels. While these effects might seem like opposing factors in the development of MetS, some observational studies have found MetS to be more prevalent in individuals with the minor allele of these SNPs [14, 16–18]. Like rs780094, rs780093 is also a common intronic variant in the GCKR gene that has been associated with polygenic dyslipidemia and high TG levels [19].

In this work, variants in the GCKR gene, as well as clinical and demographic measures, will be utilized to build predictive models for metabolic syndrome. Recently, researchers have used various machine learning algorithms to predict MetS. Methods such as decision tree, Random Forest [20, 21], and support vector machines (SVM) [22], among others, have achieved high performance in evaluations. Each algorithm has its strengths and weaknesses that might suit a particular data and question type. Here, we aimed to compare certain machine learning models (decision tree, Random Forest, support vector machines) with traditional statistical models (logistic regression, linear and quadratic discriminant analysis) developed on data from the participants of the Tehran Cardio-metabolic Genetic Study. We used models to obtain the most critical variables in predicting metabolic syndrome and finding the high-performing ones in classifying individuals regarding MetS.

## Method

### Overview and study population

Subjects for this work were selected from the Tehran Lipid and Glucose Study (TLGS). Research Institute for Endocrine Sciences (RIES) affiliated with Shahid Beheshti University of Medical Sciences, approved the study protocol and initiated TLGS in 1999. It is a dynamic cohort experiment that aims to study the risk and protective factors of non-communicable diseases in the Iranian population. 15,005 people from district 13 of Tehran have been recruited and followed through 6 phases [23]. Tehran cardio-metabolic genetic study (TCGS) is a prospective family-based cohort study within TLGS that aimed to create a comprehensive genome-wide database of the Tehranian population. Participants have been followed every three years, and at each phase, all the participants have signed written consent. Through the six phases of this study, genotype and phenotype data on 13,399 individuals have been gathered. Details on all aspects of this project, including the design and practical methods (phenotyping, genotyping, and quality controls) have been described elsewhere by Azizi F. et al. [24–26].

**Fig. 1:**
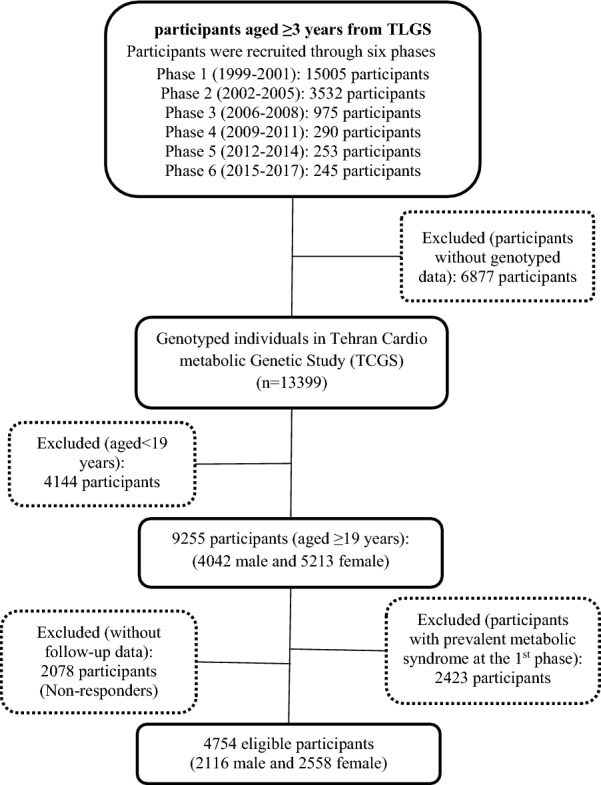
Study design and participant selection flowchart; 4754 eligible participants with available genotype information, > 19 years old, without prevalent MetS at the 1st phase and complete follow-up data from Tehran Cardio-metabolic genetic study (TCGS) were included

Of 15,005 participants who entered at the 6 phases of TLGS (1999–2017), 13,399 subjects were genotyped and were included in TCGS. From this population, for this candidate gene study, all people over 18 who were not diagnosed with MetS at the first phase were included. The following were excluded: people with missing genotyping information; participants younger than 19 years old; Participants who were prevalent cases of MetS at the first phase; participants whose baseline or follow-up data were not available; and individuals who did not consent to participate. Ultimately 4754 eligible participants (2116 men and 2558 women) were selected for this work. A detailed flowchart of patient recruitment can be viewed in Fig. 1.

### Definition of terms

For the purposes of this work, metabolic syndrome (MetS) is defined with the joint interim statement (JIS) criteria [27], that is: the presence of at least 3 of the 5 following metabolic risk factors: (1) Hypertension as DBP ≥ 85 and SBP ≥ 130 mmHg, or antihypertensive medication; (2) Fasting HDL < 40 mg/dL and < 50 mg/dL in males and females respectively, or under lipid-lowering medication; (3) Fasting serum TG ≥ 150 mg/dL or under lipid-lowering medication; (4) Fasting plasma glucose (FPG) ≥ 100 mg/dL, or taking diabetes medication; (5) and central obesity (waist circumference (WC) ≥ 90 cm for both genders, based on the Iranian National Committee for Obesity guidelines). Based on the JIS criteria, individuals with at least three metabolic risk factors were considered as unhealthy cases. Others with a maximum of two from the mentioned risk factors were deemed healthy controls. Smoking status was categorized as never, former smoker, current smoker, and second-hand smoker. For Marital status, four categories were defined: single, married, widowed, and divorced.

### Genetic analysis

Genomic DNA samples were extracted from the buffy-coat of venous blood samples using the standard proteinase K/salting out method. For qualitative estimation of the extracted DNA, a Thermo Scientific NanoDrop 1000 Spectrophotometer was used, and samples with low quality and concentration (DNA purification in the range of 1.7 < A260/A280 < 2) were excluded. DNA samples were genotyped with HumanOmniExpress-24-v1-0 bead chips (containing 649,932 SNP loci with an average mean distance of 4 kb) by deCODE genetics, Inc. (Reykjavik, Iceland) according to the manufacturer's specifications (Illumina Inc., San Diego, CA, USA). The PLINK program (V 1.07) and the R statistical software (V 3.2) used quality control procedures. The genotyping data of GCKR polymorphisms (rs780094, rs1260326, and 780,093) were used for association analysis.

### Statistical analysis

To find the essential predictors associated with metabolic syndrome, we compared classification machine learning (ML) algorithms, including the Random Forest (RF), Decision tree (DT), and Support vector machines (SVM), with three traditional statistical models: Logistic regression (LR), Linear discriminant analysis (LDA), and Quadratic discriminant analysis (QDA). The performance evaluation metrics are also reported by gender. All statistical analytical methods were performed using previously developed "randomForest", "MASS", "PRROC", "rpart", "caret",”e1071″ R packages [28–34].

### Logistic regression

Logistic regression (LR), a standard classification method, models the probability of one of the two classes of a dichotomous outcome. Here, the linear combination of predictors is linearly fitted to the response variable's mean with a binomial distribution under the logit link function.

$${\text{log}}\left( {\frac{{\text{p}}}{{1 - {\text{ p}}}}} \right) = {\upalpha } + \mathop \sum \limits_{{{\text{i}} = 1}}^{{\text{k}}} {\upbeta }_{{\text{i}}} {\text{x}}_{{\text{i}}} { }$$

P is the probability that a person has MetS, ${\upalpha }$ is the intercept. Xs denote the covariates (age, sex, schooling years, BMI, smoking status, marital status, physical activity, and SNP information of GCKR genotypes), and Bs represent regression coefficients.

### Discriminant analyses (Linear and Quadratic)

Discriminant analysis is one of the oldest classifiers first proposed by Fisher and is currently used in two major frameworks: Linear and Quadratic. These algorithms are based on the Bayes theorem and are different from LR in the classification task. These classifiers model the distribution of the independent variables (X) separately in each response class. They then use the Bayes theorem to estimate the probability of the X values' response levels. While linear discriminant analysis (LDA) computes the discriminant scores by finding the linear combination of independent variables that model and classify the response variable, the quadratic discriminant analysis (QDA) classifies the response variable with a non-linear combination of the predictors [35]. "MASS" package in R software was used to implement discriminant analyses [29].

### Decision tree

A decision tree (DT) is a supervised machine learning method used for regression and classification purposes [28]. DT predicts the target variable's value by learning simple rules represented by a decision tree. It includes three components: nodes, branches, and leaves. This algorithm classifies each sample by sorting them down the tree from the root to some leaf node. Each node in the tree specifies a test of a particular sample attribute, and each branch descending from that node corresponds to one of the possible values for this attribute. Each leaf represents the predicted value of the target variable given the values of the variables defined by the path from the root [36]. "rpart" package in R software was used to implement the decision tree algorithm [33].

### Random forest

Random forest (RF) is an ensemble-based learning algorithm Breiman [39] proposed first. It can be used for classification, regression, and unsupervised learning [28]. This algorithm is a set of non-pruned trees (classification trees based on the decision tree algorithm), and each tree is obtained by a recursive partitioning algorithm [37]. The algorithm for constructing an RF model with *T* trees from a dataset with *n* observations and p variables is as follows: (i) By the bootstrap method, a random sample with replacement with *n* number of observations is selected. (ii) A tree is created using the recursive partitioning algorithm for each sample. In each node, separation (partitioning) is performed based on a random sample of *m* number of predictive variable p. (iii) The recursive partitioning algorithm continues until the tree reaches its maximum size (i.e. terminal leaf node for each observation) without pruning the tree. (iv) the algorithm then iterates through the samples, and for each bootstrap sample, steps 1–3 are repeated. The final output will be the mode of classes for classification tasks and the average of predictions for regression analyses [38]. Common choices for *T* are 1000 trees and for *m* is $\sqrt{p}$ or log(p) [39]. Interpreting the Random Forest model can be challenging, so we need to summarize the information generated using quantitative indicators such as the variable importance (VI). VI is an indicator used to rank the predictor variables based on their influence on the response variable. The most famous indices are the Gini and permutation. The "randomForest" package in R software was used to implement this algorithm [28].

### Support vector machines

A support vector machine is another common supervised learning algorithm proposed by Vapnik to deal with classification and regression analysis [40]. It is mainly used for binary classification problems and applies to linear and non-linear data classification tasks. SVM's goal is to find the best classification function to discriminate between the two classes present in the data set. SVM creates a hyperplane or multiple hyperplanes in a high-dimensional space. The best hyperplane optimally divides the data into different classes with the maximum separation and gap between the classes (highest margin). In its non-linear classification method, SVM utilizes various kernel functions (i.e., linear, polynomial, radial basis, and sigmoid) to estimate and maximize the hyperplane margins. "e1071" package in R software was used to implement the SVM algorithm [32].

### Model assessment (validation and comparison of the models)

To evaluate the model performance more precisely and decrease the potential variance between the estimates, we utilized 10-repeated tenfold cross-validation [41]. This procedure divides the data into 10 subsets, and each subset is used to evaluate the model exclusively trained on the other nine remaining subsets. The estimates of performance obtained from 10 repeated cross-validation are then averaged to get the overall performance indices such as sensitivity (SE), specificity (SP), accuracy (ACC), the area under the receiver operating characteristics curve (AU-ROC) and kappa. It is important to note that each subset's proportion of cases and controls was held the same. Each subset properly represented the main sample and the status of the underlying community.

For each evaluation task, a confusion matrix was drawn. Evaluation metrics were defined as follows: Sensitivity indicates the proportion of patients with MetS that the algorithms correctly classify as MetS positives. Specificity indicates the proportion of healthy subjects that the algorithms correctly classifies as MetS negatives. Accuracy is the proportion of subjects among all participating individuals who were correctly classified as positives or negatives.

$$Sensitivity = \frac{TP}{{TP + FN{ }}} , \,\, Specificity = \frac{TN}{{FP + TN{ }}}$$

$$Accuracy = \frac{TP + TN}{{TP + FP + TN + FN{ }}}$$

The receiver operating characteristic (ROC) curve is another useful indicator of model performance [41]. The X-axis and Y-axis of the ROC curve are sensitivity and 1-specificity, respectively [42]. The area under the ROC curve (AU-ROC) indicates the model's discriminative ability, and its values range from 0.5 to 1. The precision-recall curve can summarize the information prediction performance with a single value as with ROC curves. This summary statistic is referred to as the AUC-PR; area under the (precision-recall) curve. By and large, the higher the AUC-PR score, the better a classifier performs on a particular task. Values closer to one indicate higher model performance. "PRROC" and "caret" R packages were used to obtain relevant performance metrics [30, 31, 34].

## Results

### Study population characteristics

Of 4754 subjects in this study, 54.8% were female, and the participants' mean age was 36.78 ± 13.21 years. Based on the JIS criteria, 2365 (50.6%) participants had MetS. Information on independent variables consisting of age, sex, schooling years, BMI, smoking status, marital status, physical activity, and SNP information of GCKR genotypes are described in Table 1. Here, the univariate p-values, calculated to compare the MetS positive and MetS negative groups on each predictor, are also presented.

**Table 1:** Comparing independent demographic and genetic predictors of MetS in the healthy and unhealthy groups

Variables	Unhealthy (MetS) (%)	Healthy (No MetS) (%)	P value
Group size (%)	2365(50.6)	2309(49.4)	
Age (mean ± SD)	40.53 ± 12.93	33.04 ± 12.47	< 0.001^a^
Schooling years (mean ± SD)	9.19 ± 4.34	10.41 ± 4.63	< 0.001^a^
BMI (mean ± SD)	27.08 ± 4.09	23.8 ± 3.95	< 0.001^a^
Physical activity (mean ± SD)	575.16 ± 923.29	452.48 ± 808.96	< 0.001^a^
Sex (%)			
Male	1249(52.81)	867 (37.55)	< 0.001^b^
Female	1116 (47.19)	1442 (62.45)	
Smoking status (%)			
Never	1213(51.29)	1323(55.94)	< 0.001^b^
Former smoker	146(6.17)	73(3.09)	
Current smoker	336 (14.21)	259 (1095)	
Second hand smoker	670 (28.33)	654 (27.65)	
Marital status (%)			
Divorced	24 (1.01)	19 (0.80)	< 0.001^b^
Married	1967 (83.17)	1612 (68.16)	
Single	312(13.19)	652(27.57)	
Widowed	62(2.62)	26 (1.10)	
rs1260326 (%)			
CC	662(27.99)	734 (31.04)	< 0.01^b^
TC	1156(48.88)	1090 (46.09)	
TT	547(23.13)	485 (20.51)	
rs780094 (%)			
CC	675 (28.54)	752 (31.80)	< 0.01^b^
TC	1156 (48.88)	1079 (45.62)	
TT	534 (22.58)	478 (20.21)	
rs780093 (%)			
CC	668(28.25)	735(31.08)	< 0.01^b^
TC	1143(48.33)	1107(46.81)	
TT	554(23.42)	476(20.13)	

*MetS* Metabolic Syndrome, *BMI* Body Mass Index, *SD* Standard Deviation; significant difference were observed in SNP information of GCKR genotypes and independent variables between healthy and unhealthy participants

^a^Student's-t test

^b^chi-square test

For both genders, baseline characteristics and common SNPs of GCKR genotypes for study participants and non-responders of the TCGS population are shown in Table 2. Based on the results, there were no significant differences between responders and non-responders in males and females other than higher BMI and lower physical activity in male non-responders and different smoking and marital status distribution between female responders and non-responders.

**Table 2:** Baseline characteristics of study participants and non-responders by common SNPs of GCKR genotypes

Variables	Male			Female		
	Responders (%)	Non-Responders (%)	P value	Responders (%)	Non-Responders (%)	P value
Group size (%)	2164 (72.28)	830 (27.72)		2590 (68.28)	1203 (31.72)	
Age(mean ± SD)	39.35 ± 14.59	41.48 ± 15.31	0.0614^1^	36.25 ± 11.51	37.06 ± 14.6	0.0649^a^
Schooling years (mean ± SD)	10.02 ± 4.65	9.91 ± 4.4	0.582^1^	9.58 ± 4.4	9.32 ± 4.36	0.145^a^
BMI (mean ± SD)	24.86 ± 3.87	25.12 ± 4.38	0.0246^1^	26.31 ± 4.68	26.63 ± 4.92	0.0539^a^
Physical activity (mean ± SD)	607.79 ± 1049.11	524.49 ± 1034.92	0.0510^1^	429.78 ± 696.21	384.48 ± 682.76	0.0607^a^
Smoking status (%)						
Never smoker	825 (38.12)	283 (34.10)	0.221^2^	1732 (66.87)	717 (59.60)	0.002^b^
Former smoker	219 (10.12)	88 (10.60)		8 (0.31)	13 (1.08)	
Current smoker	547(25.28)	196 (23.61)		59 (2.28)	35 (2.91)	
Second hand	573(26.48)	171 (20.60)		791 (30.54)	371 (30.84)	
Marital status (%)						
Divorced	12 (0.55)	1(0.05)	0.104^2^	33 (1.27)	18 (1.50)	< 0.001^b^
Married	1628 (75.23)	649(29.99)		2011 (77.64)	954 (79.30)	
Single	520 (24.03)	176 (8.13)		462 (17.84)	155 (12.88)	
Widowed	4 (0.18)	3 (0.14)		84 (3.24)	76 (6.32)	
rs1260326 (%)						
CC	662 (30.59)	265 (31.93)	0.233^2^	755 (29.15)	370 (30.76)	0.602^b^
TC	1015 (46.90)	402 (48.43)		1265 (48.84)	574 (47.71)	
TT	487 (22.50)	163 (49.64)		570 (22.01)	259 (21.53)	
rs780094 (%)						
CC	676 (31.24)	251 (30.24)	0.30^2^	773 (28.30)	358 (29.76)	0.998^b^
TC	1015 (46.90)	414 (49.88)		1256 (48.49)	584 (48.55)	
TT	473 (21.86)	165 (19.88)		561 (21.66)	261 (21.70)	
rs780093 (%)						
CC	672 (31.05)	179 (21.57)	0.176^2^	754 (29.11)	280 (23.28)	0.087^b^
TC	1010 (46.67)	315 (37.95)		1275 (49.23)	404 (33.58)	
TT	482 (22.27)	125 (15.06)		561 (21.66)	214 (17.79)	

*BMI* Body Mass Index; There were no significant differences between responders and non-responders in males and females other than higher BMI and lower physical activity in male non-responders and different smoking and marital status distribution between female responders and non-responders

^a^Student's t-test

^b^chi-square test

We calculated adjusted OR value and its corresponding significance level for each predictor based on the logistic regression model. The results showed that metabolic syndrome was significantly associated with age, gender, schooling years, BMI, physical activity, rs780094, and rs780093 (P < 0.05) (Table 3). Males were 2.373 times more at risk to have metabolic syndrome than females. The odds of developing metabolic syndrome decreases with increasing education years (OR = 0.978). On GCKR polymorphisms (rs780094, rs1260326, and 780,093), the results showed that MetS is associated with rs780094 and rs780093 and this relationship is caused by significantly more frequency of minor T alleles in patients with MetS.

**Table 3:** Applying logistic regression to assess the significance of relationship between Independent demographic and genetic variables and metabolic syndrome

Variables		B	Odds ratio (OR)	P value
Age		0.025	1.025	< 0.001
Gender (female = 0)		0.864	2.373	< 0.001
Schooling years		− 0.021	0.978	0.009
BMI		0.207	1.230	< 0.001
Physical activity		0.0001	1.000	0.005
Smoking status	Current smoker(reference)			
	Never smoker	0.005	1.005	0.962
	Former smoker	0.072	1.075	0.698
	Second hand	0.646	1.066	0.593
Marital status	Divorced (reference)			
	Married	− 0.087	0.916	0.808
	Single	− 0.198	0.820	0.595
	Widowed	− 0.010	0.989	0.981
rs1260326	CC(reference)			
	TC	0.206	1.229	0.347
	TT	0.472	1.603	0.133
rs780094	CC(reference)			
	TC	0.149	1.161	0.664
	TT	− 1.211	0.298	0.008
rs780093	CC(reference)			
	TC	− 0.122	0.884	0.664
	TT	1.066	2.903	0.002

*BMI* Body Mass Index; logistic regression is used to predict the metabolic syndrome status of the participants in TCGS. The metabolic syndrome was significantly associated with age, gender, schooling years, BMI, physical activity, rs780094, and rs780093 (P < 0.05)

### Performance comparison between machine learning algorithms

Table 4 summarizes the classification performance of various machine learning and traditional statistical methods based on the average value of 10-repeated tenfold cross-validation overall and by gender. Overall, the Random Forest showed higher classification accuracy (mean = 0.743) and area under the ROC curve (mean = 0.804) and AUC-PR (mean = 0.776). The decision tree ranked second in overall accuracy (mean = 0.738) with relatively high specificity (mean = 0.804) and AUC-PR (mean = 0.730). By and large, machine learning algorithms provided better accuracy, AUC-ROC, and AUC-PR compared with traditional statistical models. Accuracy, kappa, and AUC-ROC and AUC-PR of the machine learning models were higher overall and in both genders. While linear discriminant analysis (LDA) showed a high sensitivity (mean = 0.915), its specificity was considerably lower (mean = 0.230). Similar to the overall results, LDA provided the highest sensitivity in males (mean = 0.754). But in females, the highest sensitivity was achieved through logistic regression (mean = 0.798).

**Table 4:** Performances metrics for LR, SVM, DT, RF, LDA, and QDA algorithms

	Models	Accuracy	Sensitivity	Specificity	Kappa	AUC-ROC	AUC-PR
Total	SVM	0.725	0.661	0.785	0.447	0.785	0.761
	DT	0.738	0.667	0.804	0.473	0.771	0.730
	RF	0.743	0.699	0.784	0.484	0.804	0.776
	LR	0.705	0.677	0.732	0.409	0.770	0.748
	LDA	0.562	0.915	0.230	0.141	0.658	0.666
	QDA	0.546	0.492	0.598	0.089	0.563	0.555
Male	SVM	0.712	0.475	0.870	0.366	0.733	0.766
	DT	0.735	0.527	0.874	0.421	0.739	0.753
	RF	0.729	0.559	0.842	0.415	0.754	0.782
	LR	0.711	0.519	0.839	0.373	0.732	0.768
	LDA	0.591	0.754	0.482	0.217	0.679	0.734
	QDA	0.547	0.394	0.649	0.044	0.531	0.616
Female	SVM	0.733	0.783	0.671	0.456	0.802	0.565
	DT	0.748	0.753	0.742	0.492	0.785	0.706
	RF	0.744	0.767	0.715	0.482	0.815	0.754
	LR	0.738	0.798	0.663	0.465	0.803	0.741
	LDA	0.608	0.752	0.427	0.184	0.664	0.617
	QDA	0.635	0.749	0.491	0.245	0.670	0.572

*LR* Logistic Regression, *SVM* support vector machines, *DT* Decision Tree, *RF* Random Forest, *LDA* Linear discriminant analysis, *QDA* Quadratic discriminant analysis, *AUC* Area Under Curve. Machine learning methods outperforms the traditional statistical methods

**Fig. 2:**
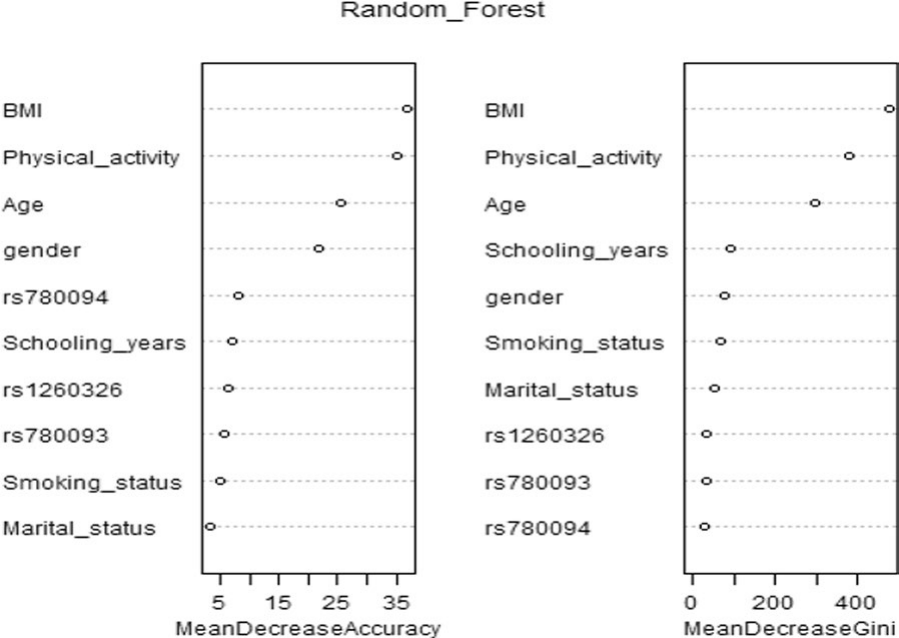
Assessing the importance of predictors with Gini and Accuracy importance indices based on the implementation of the random forest model; we confirmed that BMI, physical activity, and age were the most influential variables in MetS prediction

The importance of variables in the Random Forest model was calculated using the mean decrease Gini and mean decrease accuracy and is shown in Fig. 2. BMI, physical activity, and age were the most influential variables in both indices.

**Fig. 3:**
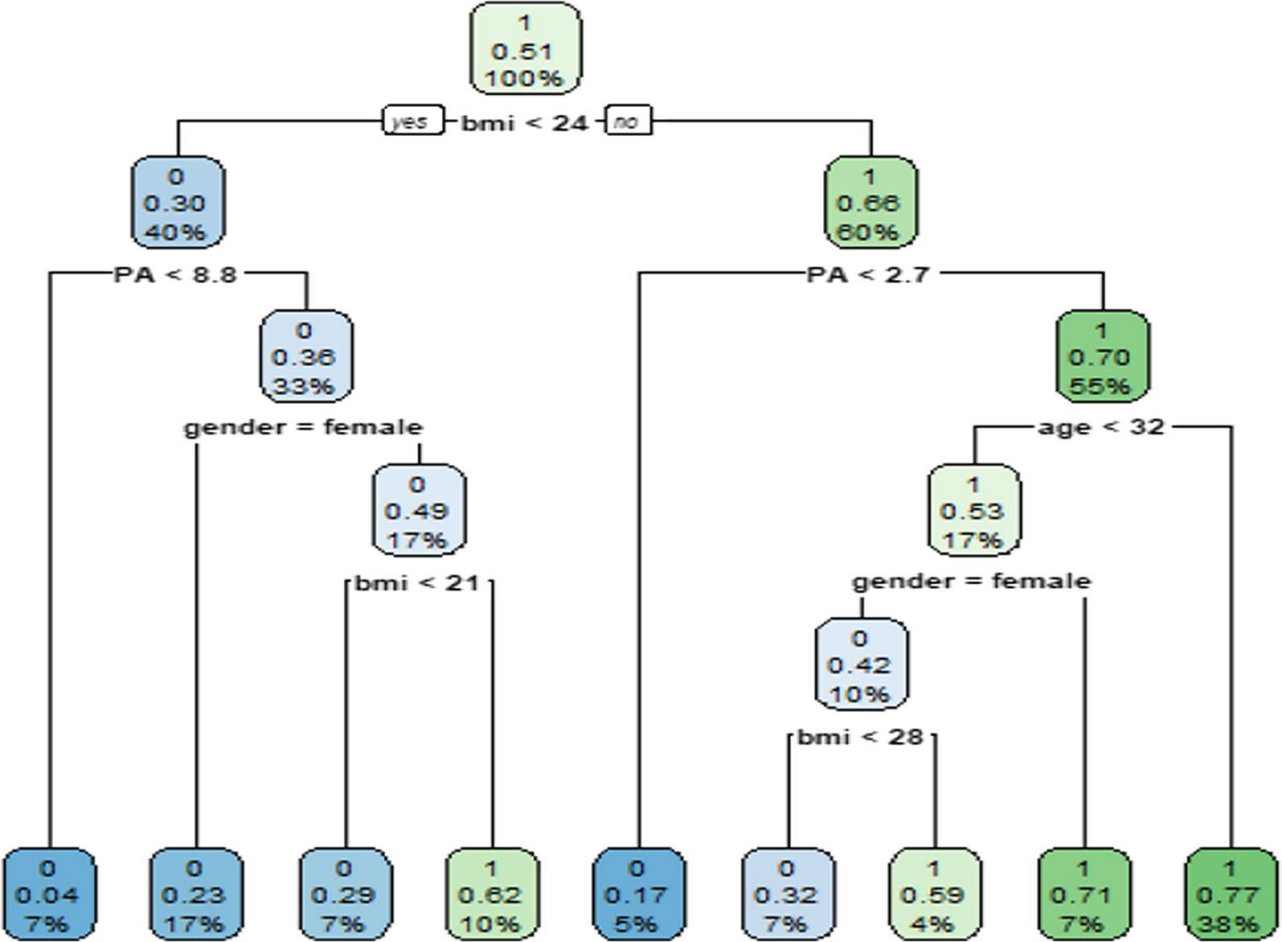
Classification decision tree, with probabilities of success for metabolic syndrome shown in each node; A combination of BMI, Physical activity, and age is an accurate predictor for the MetS

BMI present in the tree root was the most significant decision tree method and acted as the main prognostic factor. A combination of BMI + Physical activity + age is an accurate predictor for MetS. According to the induced decision tree shown in Fig. 3, the probability that an individual with BMI < 24 and physical activity < 8.8 has MetS is a mere 4%. In contrast, there is a 77% probability that a person with BMI ≥ 25, physical activity < 2.7, and age ≥ 33 suffers from MetS.

## Discussion

This study aimed to compare the performance of machine learning-powered classification models, namely, support vector machines (SVM), decision tree (DT), and Random Forest (RF) in predicting metabolic syndrome, to that of three traditional classifiers: logistic regression (LR), linear discriminant analysis (LDA), and quadratic discriminant analysis (QDA). Through developing such models on the eligible participants of the Tehran Cardio-metabolic genetic study (TCGS), we also obtained the most influential predictive features of MetS among clinical and GCKR polymorphism variables.

We found age, gender, schooling years, BMI, physical activity, and genetic variants of rs780094 and rs780093 significant risk factors for predicting metabolic syndrome. Despite their statistical significance, gender, schooling years, rs780094, and rs780093 did not influence the MetS prediction considerably. On the other hand, BMI, physical activity, and age were the most influential predictors of MetS, as indicated by the influence metrics of the Random Forest model. This result is in line with Fuentes et al., which denoted BMI as one of the anthropometric variables associated with metabolic syndrome and essential for early detection [43].

The single nucleotide polymorphisms that showed significant relationships with metabolic syndrome in our predictive models agree with the findings of previous works that had examined the association between MetS and similar genetic markers [18, 44, 45].

Among classification machine learning methods that included SVM, DT and RF, RF had the best performance in classifying subjects on their MetS outcomes as indicated by the highest accuracy (0.743) as well as area under the receiver operating characteristic curve (AU-ROC) (0.804) and AUC-PR (0.776). This result is similar to the findings in the study by Szabo et al. that applied the Random Forest algorithm for a similar task and calculated the accuracy of this method to be 71.4% [46–48]. Worachartcheewan et al. also implemented a Random Forest model to predict MetS in the Bangkok population and identify the most influential predictors. They found that the Random Forest algorithm predicted MetS status in adults aged 18 to78 with high accuracy (98.11%) [49].

The decision tree was the second-best performing model, and its calculated measure for accuracy, sensitivity, specificity, AUC-ROC, and AUC-PR were 0.738, 0.667, 0.804, 0.771, and 0.730, respectively. Other works have also implemented the decision tree to detect metabolic syndrome with a sensitivity of 91.6% and specificity of 95.7% [43]. Results obtained from the decision tree algorithm showed that a combination of BMI, physical activity, and age is an accurate predictor for predicting MetS. This agrees with a previous work by Huang et al. conducted to explore the association between lifestyle variables and metabolic syndrome and found that individuals with BMI > 27 kg/m^2^ were predisposed to metabolic syndrome [50]. In another study by Worachartcheewan et al. that used a decision tree to diagnose metabolic syndrome, the results confirmed that BMI ≥ 25 is an important feature in diagnosing MetS [20]. In our work, the evaluation metrics for DT were almost similar to RF, and both outperformed the SVM. Karimi-Alavijeh et al. also employed DT and SVM to predict metabolic syndrome. In that investigation, SVM outperformed DT on several performance metrics (SVM (DT) model accuracy, sensitivity, and specificity were 0.774 (0.758), 0.74 (0.72), and 0.757 (0.739) [51].

To construct predictive models for MetS, other works have similarly employed various data mining methods including artificial neural networks (ANN), beside decision tree, Random Forest, support vector machines, principal component analysis (PCA) and association analysis (AA). Their results showed that with an accuracy in the north of 99%, DT outperformed ANN and SVM, which provided lower accuracy metrics [52]. Other investigators have shown DT to be a robust machine learning method for constructing a predictive model of metabolic syndrome with reported accuracies of 73.90% [53] and 71.80% [54]. Lin et al. attempted to identify MetS in patients undergoing treatment with second-generation antipsychotics. They reported that logistic regression model had an accuracy as high as 83.6% and indicated BMI was an important predictor in identifying metabolic syndrome status [54]. This result contrasts with studies that found RF and SVM to be the most accurate classifiers for metabolic syndrome [22, 28, 55]. The complicated and multifactorial nature of metabolic syndrome and the severity of its complications require investigators to put further emphasis on the model sensitivity. While the quadratic discriminant analysis provided very low sensitivity, linear discriminant analysis (LDA) had the highest overall sensitivity. Similar to ours, other investigations have shown that LDA and RF are more sensitive classifiers than SVM, classification tree, and ANN [22, 56–58].

Compared with other recent studies conducted to develop predictive models for MetS, this work provides several advantages. It is important to emphasize that metabolic syndrome is a multifactorial disorder in which genetics, environmental factors, and lifestyle habits are all involved in disease pathogenesis. Unlike studies that exclusively use genetic variables, we developed our predictive models using clinically important and genetic information to provide more relevant results. In addition, past modeling efforts have less frequently developed both traditional and machine learning algorithms on big data for MetS prediction. The machine learning models developed through this effort have the advantage of providing good patient classification and indicating the most important risk factors. These models can be the basis of clinical tools that receive genetic and environmental information from patients as inputs and output their chances of having/developing MetS.

On the other hand, we should emphasize that researchers should be cautious when generalizing the results of this effort to other populations that were not represented in our study sample. Moreover, the effect of slight differences between responders and non-responders among participants on the study metrics remains unclear.

## Conclusion

It is essential to focus the resources on individuals who are most likely to develop or be already afflicted with these disorders to improve the potential effects of public health measures in reducing the burden of prevalent diseases such as metabolic syndrome. Traditional statistical models often fail to provide reliable predictive models when facing a multifactorial disorder with many potential independent genetic and environmental risk factors. However, as compared to conventional models, modern machine learning algorithms can enhance predicted accuracy in clinical concerns. Nonetheless, even when combined with genetic information, these models are insufficient for clinical application [59]. The first reason is that the sample size of such studies is insufficient to make a conclusive determination; the second reason is that whole-genome information is required in this regard; additionally, the ancestral discrepancy between populations necessarily requires that these models be considered separately for different ethnic groups [60, 61]. In this work, we compared predictive models for metabolic syndrome using the information on demographic and clinical and genetic data (functional variants of GCKR gene) on patients from TCGS. Our results proved modern methods, particularly Random Forest and decision tree, can provide high performing MetS predicting models that can help reduce future cardiovascular, cancer, or other related complications when integrated within decision support tools or future investigations.

The study was the first step to predict the phenotypes using the polygenic risk score (PRS) as a modern method for disease prediction. The vital thing in the TCGS is discovering the best prediction model(s) for different diseases, especially MetS, which is multifactorial in terms of the definition and the etiology. Consequently, we decided to test the conventional models versus machine learning methods for known genes in our data to compare them on prediction ability.

## Supplementary Information

**Additional file 1**. Readers can refer to the supplementary material file to see more details of the accuracy indices of the models for each repeat and each fold.

## Data Availability

The datasets generated and/or analysed during the current study are not publicly available due to containing information that could compromise the privacy of research participants but are available from the corresponding author on reasonable request.

## Abbreviations

AA: Association analysis
ACC: Accuracy
ANN: Artificial neural networks
AUC: Area under the curve
AU-ROC: Area under the receiver Operating characteristics
AUC-PR: Area under the precision-recall curve
BMI: Body Mass Index
CVD: Cardiovascular diseases
DBP: Diastolic Blood Pressure
DT: Decision tree
FN: False negative
FP: False positive
FPG: Fasting plasma glucose
GCK: Glucokinase
GCKR: Glucokinase regulator
GKRP: Glucokinase regulatory protein
GWAS: Genome-wide association studies
HDL: High-density lipoprotein
JIS: Joint interim statement
LDA: Linear discriminant analysis
LR: Logistic regression
MetS: Metabolic syndrome
ML: Machine learning
OR: Odds ratio
PCA: Principal component analysis
PRS: Polygenic risk score
QDA: Quadratic discriminant analysis
RF: Random forest
RIES: Research Institute for Endocrine Sciences
ROC: Receiver operating characteristic
SBP: Systolic blood pressure
SD: Standard deviation
SE: Sensitivity
SP: Specificity
SVM: Support vector machines
T2D: Type 2 diabetes
TCGS: Tehran cardio-metabolic genetic study
TG: Triglyceride
TLGS: Tehran lipid and glucose study
TN: True negative
TP: True positive
WC: Waist circumference
